# A Dual-Approach Strategy to Optimize the Safety and Efficacy of Anti-Zika Virus Monoclonal Antibody Therapeutics

**DOI:** 10.3390/v15051156

**Published:** 2023-05-11

**Authors:** Haiyan Sun, Ming Yang, Huafang Lai, Biswas Neupane, Audrey Y.-H. Teh, Collin Jugler, Julian K.-C. Ma, Herta Steinkellner, Fengwei Bai, Qiang Chen

**Affiliations:** 1 The Biodesign Institute and School of Life Sciences, Arizona State University, Tempe, AZ 85287, USA; 2 Department of Cell and Molecular Biology, University of Southern Mississippi, Hattiesburg, MS 39406, USA; 3 Institute for Infection and Immunity, St. George’s, University of London, London SW17 0RE, UK; 4 Department of Applied Genetics and Cell Biology, University of Natural Resources and Life Sciences, 1180 Vienna, Austria

**Keywords:** Zika virus, monoclonal antibody (mAb), plant-made antibody, antibody-dependent enhancement of infection (ADE), antibody-dependent cellular cytotoxicity (ADCC), glycosylation, Fc effector function, plant-made pharmaceutical

## Abstract

Antibody-dependent enhancement of infection (ADE) is clinically relevant to Dengue virus (DENV) infection and poses a major risk to the application of monoclonal antibody (mAb)-based therapeutics against related flaviviruses such as the Zika virus (ZIKV). Here, we tested a two-tier approach for selecting non-cross-reactive mAbs combined with modulating Fc glycosylation as a strategy to doubly secure the elimination of ADE while preserving Fc effector functions. To this end, we selected a ZIKV-specific mAb (ZV54) and generated three ZV54 variants using Chinese hamster ovary cells and wild-type (WT) and glycoengineered ΔXF *Nicotiana benthamiana* plants as production hosts (ZV54^CHO^, ZV54^WT^, and ZV54^ΔXF^). The three ZV54 variants shared an identical polypeptide backbone, but each exhibited a distinct Fc N-glycosylation profile. All three ZV54 variants showed similar neutralization potency against ZIKV but no ADE activity for DENV infection, validating the importance of selecting the virus/serotype-specific mAbs for avoiding ADE by related flaviviruses. For ZIKV infection, however, ZV54^CHO^ and ZV54^ΔXF^ showed significant ADE activity while ZV54^WT^ completely forwent ADE, suggesting that Fc glycan modulation may yield mAb glycoforms that abrogate ADE even for homologous viruses. In contrast to the current strategies for Fc mutations that abrogate all effector functions along with ADE, our approach allowed the preservation of effector functions as all ZV54 glycovariants retained antibody-dependent cellular cytotoxicity (ADCC) against the ZIKV-infected cells. Furthermore, the ADE-free ZV54^WT^ demonstrated in vivo efficacy in a ZIKV-infection mouse model. Collectively, our study provides further support for the hypothesis that antibody–viral surface antigen and Fc-mediated host cell interactions are both prerequisites for ADE, and that a dual-approach strategy, as shown herein, contributes to the development of highly safe and efficacious anti-ZIKV mAb therapeutics. Our findings may be impactful to other ADE-prone viruses, including SARS-CoV-2.

## 1. Introduction

The Zika virus (ZIKV) is an arbovirus that is closely related to other members of the *Flavivirus* genus such as the dengue virus (DENV), West Nile virus (WNV), Japanese encephalitis virus (JEV), yellow fever virus (YFV), and tick-borne encephalitis virus (TBEV) [1]. Most ZIKV infections in humans have been sporadic in Africa and associated with only mild febrile illnesses prior to the large outbreak in the Pacific and the Americas [2]. The new emergence of ZIKV in French Polynesia and the Americas is associated with a dramatic increase in human infections with severe congenital malformations including microcephaly in neonates and neurological complications in adults, such as Guillain-Barré syndrome [3,4]. In the absence of licensed vaccines [5], the high potential for more wide-spread outbreaks in tropical and urban areas calls for the development of effective and safe therapeutics to treat ZIKV infection.

Monoclonal antibodies (mAbs) against the envelope (E) protein are a class of biologics with strong potential as effective ZIKV therapeutics. Results from flavivirus vaccine studies and clinical trials have provided strong support for such a hypothesis. For example, in animal models, neutralizing antibody responses have been found to be the major correlate of protection for licensed vaccines against YFV and TBEV, along with providing protection against infection by many other flaviviruses [6,7,8]. Similar to other flaviviruses, the E protein of ZIKV mediates the viral entry process and has a typical three-domain structure (EDI, EDII, and EDIII) [9,10,11]. The E proteins of flaviviruses are major targets of host humoral responses [1,9], and antibodies in naturally infected patients or in subjects vaccinated with E protein-based antigens have been mapped to all three E domains [8,12]. A majority of the E-specific antibodies in infected humans are against the epitopes on the EDI and EDII domains of the E protein. Some of these antibodies are neutralizing, with most of them being cross-reactive among different serotypes/strains of various flaviviruses [13,14]. In contrast, antibodies mapped to EDIII are typically virus- or serotype-specific due to the lower sequence similarity of EDIII among various flaviviruses compared to EDI and EDII [15].

One of the major impediments of deploying mAbs as therapeutics against ZIKV infection is the risk of antibody-dependent enhancement (ADE). ADE has been shown to be clinically relevant to DENV infection [16]. It is hypothesized that when patients encounter a new serotype of DENV after a first infection, some of the cross-reactive antibodies from the primary infection may enhance viral entry and replication in myeloid cells by forming immune complexes and trigging Fc gamma receptor (FcγR)-mediated endocytosis [16,17]. This may lead to hyperinflammatory responses and cytokine storms, potentially causing lethal dengue hemorrhagic fever/dengue shock syndrome (DHF/DSS) [16,18,19]. Antibodies against the ZIKV E protein also carry the risk of exacerbating DENV symptoms via ADE as the E proteins of the two flaviviruses share many common epitopes [20,21]. Indeed, the ADE of DENV infection and disease severity through anti-ZIKV antibodies have been demonstrated both in vitro and in vivo [15,22,23,24,25]. Therefore, mAb therapeutics for ZIKV must be evaluated for both their therapeutic potency and risk of ADE because enhancement of DENV infection—not just a lack of protection against ZIKV—is of concern.

Here, we used ZV54, a non-cross-reactive mAb recognizing a unique epitope on the lateral ridge of ZIKV EDIII (zDIII) [8], as a model mAb. We investigated if the combination of selecting virus/serotype-specific mAbs and Fc glycan modulation can be used to doubly ensure the elimination of ADE while preserving beneficial Fc-effector function. Three glycovariants of the ZV54 mAb were generated by producing the mAb in Chinese hamster ovary (CHO) cells and in wild-type (WT) and glycoengineered ΔXF *Nicotiana benthamiana* plant lines. The ΔXF is a mutant *N. benthamiana* line in which the transferases for producing plant-specific xylose and fucose N-glycans have been knocked down by a stable RNAi mechanism [26]. The three ZV54 mAb glycovariants carried distinct N-glycosylation profiles with plant-derived ZV54 exhibiting one predominant glycoform and CHO-made ZV54 displaying glycan heterogeneity. The ZV54 glycovariants specifically recognized the zDIII antigen and effectively neutralized live ZIKV with similar potency. However, their ADE activity is intriguing. All three glycovariants forwent ADE activity for DENV infection as ZV54 was specifically selected for its lack of cross-reactivity with other flaviviruses. Furthermore, while the ADE activity for ZIKV infection was observed for the CHO- and glycoengineered plant-produced ZV54 variants, the ZV54 glycoform derived from WT plants displayed no ADE activity. Interestingly, the antibody-dependent cellular cytotoxicity (ADCC) against ZIKV-infected cells was preserved for all three ZV54 glycoforms, with the plant-made glycoforms exhibiting higher activity. Further analysis demonstrated the in vivo therapeutic efficacy for the ADE-free ZV54 mAb glycovariant in a mouse model.

## 2. Material and Methods

### 2.1. Ethics Statement

All animal experimental procedures were approved by the Institutional Animal Care and Use Committees (IACUC # 16031002) of the University of Southern Mississippi (USM). The in vivo and in vitro experiments with live ZIKV and DENV were performed in biosafety level 2 (BSL2) laboratories at the USM and Arizona State University (ASU) by certified researchers who followed the standard biosafety protocols that have been approved by the USM and ASU Institutional Biosafety Committees (IBC).

### 2.2. Gene Constructs for ZV54 mAb Expression in N. benthamiana Leaves and Chinese Hamster Ovary Cells

The genes for the variable regions of the heavy chain (HC) and light chain (LC) of ZV54 [8] were synthesized and cloned into vectors containing the coding sequence of the human IgG1 HC constant region and the kappa LC constant region, respectively [27]. The resulting HC and LC genes were cloned into MagnICON-based plant expression vectors and transformed into *A. tumefaciens* as described previously [28]. The *Agrobacterium* strains harboring the ZV54 HC and LC 3′ vectors were agroinfiltrated into the leaves of 6-week-old WT and ∆XF *N. benthamiana* plants as we described previously [29,30,31]. For the CHO cell expression, the coding sequences of HC and LC were cloned into the mammalian cell expression vector pcDNA3.1 (Thermo Fisher Scientific, Waltham, MA, USA) and transfected into CHO-K1 cells with lipofectamine (Thermo Fisher Scientific, Waltham, MA, USA) according to the manufacturer’s protocol [32].

### 2.3. Production and Purification of ZV54 mAb from N. benthamiana Plants and CHO Cells

A sandwich ELISA that detected only the assembled form of IgG was used to evaluate the temporal expression patterns of the ZV54 mAbs in *N. benthamiana* leaves as previously described [32]. Briefly, the leaves were harvested 5, 6, 7, and 8 days post-vector-infiltration (DPI) and then homogenized in extraction buffer (50 mM of Tris-HCl, pH 7.5, and 150 mM of NaCl) to obtain the total leaf soluble protein. The leaf extracts were clarified by centrifugation at 15,000× *g* for 30 min at 4 °C, and the clarified total leaf protein was transferred to the wells of microtiter plates that had been coated with a goat anti-human gamma HC antibody (Southern Biotech, Birmingham, AL, USA). The plates were washed after 1 h of incubation, and an HRP-conjugated anti-human-kappa LC antibody (Southern Biotech, Birmingham, AL, USA) was added for detection. E16 mAb, an anti-WNV DIII IgG isotype with a human IgG1 HC constant region [33], was used as a reference standard. For the other protein analyses, ZV54 was extracted from leaves at 7 DPI as described above and purified with the protein A-based method described previously [34].

For production in the CHO cells, ZV54 was directly enriched from the cell culture media by spinning at 15,000× *g* for 30 min, followed by purification from clarified media with protein A affinity chromatography using MabSelect resin (Cytiva, Marlborough, MA, USA) according to the manufacturer’s protocol.

### 2.4. Analysis of N-Linked Glycosylation of ZV54

The N-glycosylation profiles of the ZV54 variants were determined by mass spectrometry (MS) as described previously [35,36,37]. Essentially, the purified ZV54 mAbs were separated on SDS-PAGE and the HC was extracted from the gel, trypsin-digested, and analyzed with a liquid-chromatography-electrospray ionization-mass spectrometry (LC-ESI-MS) system (Orbitrap Exploris 480, Thermo Scientific, Waltham, MA, USA). Various N-glycans (including N-acetylhexosamine units, mannose, galactose, glucose, and xylose residues) and their associated peptides were detected as a set of glycopeptide peaks. The identification of the glycopeptide peaks was revealed by searching with FreeStyle 1.8 (Thermo Scientific, Waltham, MA, USA) and deconvoluting using the extract function. The glycans were annotated according to the nomenclature of the Consortium for Functional Glycomics [38].

### 2.5. Yeast Display of zDIII and Flow Cytometry

The yeast-expressing zDIII was generated and stained with ZV54 glycovariants as we described previously [33]. Briefly, the coding sequence of zDIII corresponding to amino acid residues 303-403 of the ZIKV E protein was cloned in the yeast surface display vector pYD1 (Invitrogen, Waltham, MA, USA) in which the GAL1 promoter controlled the expression of zDIII. The vector was then transformed into the *S. cerevisiae* yeast strain EBY100 (ATCC# MYA-4941). The transformed yeast was grown at 30 °C in tryptophan-free media with 2% glucose (Sigma, St. Louis, MO, USA) and harvested when it reached the log phase. The zDIII expression on the yeast’s surface was induced by growing the yeast for 24 h in the same media but with 2% galactose at 25 °C. The induced yeast was harvested, washed with PBS, and stained with the ZV54 glycovariants or the E16 isotype control mAb. After washing, the yeast was stained with an Alexa Fluor-conjugated goat anti-human secondary antibody (Molecular probes, Eugene, OR, USA) and analyzed with a Gallios flow cytometer (Beckman Coulter, Brea, CA, USA). The uninduced yeast was stained and analyzed in parallel as a negative control.

### 2.6. Surface Plasmon Resonance

The binding activities of the ZV54 variants for the human FcγRIIA and FcγRIIIA were measured by surface plasmon resonance (SPR) on a BIAcore X-100 instrument (Cytiva, Marlborough, MA, USA) at 25 °C as described previously [39]. The experiments were performed with a CM5 chip that was coated with protein A (Sigma-Aldrich, St. Louis, MO, USA) on both flow cells to 3000–4000 response units (RUs) using standard amine coupling, and HBS-EP+ (10 mm of HEPES, pH 7.4, 150 mm of NaCl, 3 mm of EDTA, and 0.05% surfactant P-20) was used as the running buffer. The ZV54 glycovariants were diluted in HBS-EP+ buffer and captured onto the protein A surface to the levels of ~330 RU (for FcγRIIIA) and 680 RU (for FcγRIIA), respectively. The recombinant human FcγR ectodomains (R&D Systems, Minneapolis, MN, USA) were injected over both flow cells at 25 °C for 40 s at 50 μL/min (FcγRIIIA, when tested against fucosylated antibodies), 90 s at 40 μL/min (FcγRIIIA, when tested against afucosylated antibodies), or 30 s at 45 μL/min (FcγRIIA). The dissociation times were 60 s and 600 s for FcγRII and FcγRIIIA, respectively. Each receptor was injected in duplicate with the following concentrations: FcγRIIIA (0.0625, 0.125, 0.25, 0.5, and 1 µM) and FcγRIIA (0.125, 0.25, 0.5, 1, and 2 µM). Between experiments, the surface was regenerated by two 60 s pulses of regeneration buffer (10 mm glycine-HCl, pH 1.5). The binding data were fitted with BIAcore Evaluation software X100 using the two-state reaction model (FcγRIIIA) or steady-state affinity (FcγRIIA) to calculate the equilibrium dissociation constant (KD). Two biological replicates were performed for each experiment.

### 2.7. Neutralization against ZIKV

Plaque reduction neutralization test (PRNT) assays were performed to examine the neutralizing activities of the ZV54 glycovariants against ZIKV as previously described [40,41]. Briefly, the ZIKV samples (PRVABC59, ATCC# VR-1843) were diluted to a working concentration of 100 plaque-forming units (PFU) per well in Opti-MEM serum-free medium (Life Technologies, Carlsbad, CA, USA). The ZIKV samples were then mixed with the ZV54 mAb glycovariants that had been serially diluted in Opti-MEM. After 1 h of incubation at 37 °C, the virus–mAb mixture was transferred to a plate with confluent Vero cells (ATCC # CCL-81). The cells were then overlaid with fresh medium containing 0.8% agarose (Invitrogen, Waltham, MA, USA) and incubated for an additional 3 days at 37 °C. The cells were subsequently fixed with 4% paraformaldehyde (PFA, MilliporeSigma, Burlington, VT, USA), followed by staining with 0.2% crystal violet (Sigma, St. Louis, MO, USA). The percent (%) neutralization was calculated using the following formula: ((number of plaques per well without mAb) − (number of plaques per well with diluted mAb)/(number of plaques per well without mAb) × 100). The half-maximal inhibitory concentration (IC_50_) of each mAb was calculated using GraphPad Prism software (Version 9.3). Each mAb glycovariant was tested in triplicate and in at least two independent experiments.

### 2.8. ADE Assay

We used an established protocol [42] to measure the ADE activities of the ZV54 glycovariants for the DENV-2 (ATCC, VR-1584) and ZIKV infections in human FcγRIIA^+^ K562 cells (ATCC # CCL-2243). The ZV54 mAb glycovariants were first serially diluted and then mixed with DENV-2 or ZIKV. After incubating for 1 h at 37 °C, the mAb-virus complexes were incubated with K562 cells (MOI = 1). The cells were collected 48 h (DENV-2) or 72 h (ZIKV) post-incubation. The cells were then fixed with 4% PFA, permeabilized with 0.1% saponin (Sigma, St. Louis, MO, USA), and stained with mAb 4G2 (ATCC# HB112) conjugated to Alexa 488 (Invitrogen, Waltham, MA, USA). The percentages of DENV- or ZKIV-infected K562 cells were determined by flow cytometry with a Gallios flow cytometer (Beckman Coulter, Brea, CA, USA).

### 2.9. ADCC Assay

Preparation of the effector cells: Human natural killer (NK) cells were expanded through a 21-day period according to a published protocol [43]. The expanded NK cells were cultured in RPMI medium (ThermoFisher, Waltham, MA, USA) with 10% fetal bovine serum (FBS, Sigma) plus 50 units/mL of human IL-2 (Sigma, St. Louis, MO, USA) for 24 h. The NK cells (50,000 cells/well) were then plated in a 96-well V bottom TC plate (ThermoFisher, Waltham, MA, USA) at density, with an E/T ratio of 5:1.

Preparation of the target cells: The Vero cells were infected with ZIKV for 4 days with an MOI of 0.05. The cells were then rinsed with PBS and dislodged using a cell scraper in PBS with 8 mM of EDTA (Sigma, St. Louis, MO, USA). The negative control uninfected Vero cells were also cultured and dislodged in parallel. The dislodged cells were resuspended in MEM media containing 5% FBS to a density of 1 million/mL and loaded with 5 µg/mL of calcein AM (Invitrogen, Waltham, MA, USA) at 37 °C for 1 h. After calcein AM-loading, the infected or uninfected Vero cells were resuspended in RPMI with 10% FBS at 0.1 million/mL and incubated with the mAb variants (20 µg/mL) or a human IgG isotype control for 15 min.

After adding the Vero cell–mAb mixture to the NK cell-containing TC plate, the assay was initiated by centrifuging the TC plate at 100× *g* for 1 min. The TC plate was centrifuged at 100× *g* for 5 min after a 4 h incubation period (at 37 °C) and the supernatant was transferred to a new 96-well black/clear bottom plate (ThermoFisher, Waltham, MA, USA). Fluorescence was determined using a 485 excitation/530 emission filter set in a SpectraMax M5 fluorometer (Molecular Device, San Jose, CA, USA). Maximum release control was measured from the supernatants of the wells that contained only Vero cells (no NK cells or mAb) and treated with 100 µL of 2% Triton X-100 (ThermoFisher, Waltham, MA, USA). Spontaneous release control was determined from the supernatants of the wells that contained only Vero cells, and they were treated with 100 µL of RPMI media. The percentage of mAb–NK-cell-mediated cell lysis was calculated using the following formula: ((test release - spontaneous release)/maximum release) × 100. Each sample was measured in triplicate, and the experiments were repeated at least twice.

### 2.10. Mouse Studies

Five-week-old type I interferon receptor-deficient (*Ifnar1*^−/−^) mice with a C57BL/6J background were purchased from the Jackson Laboratory (Bar Harbor, ME, USA) and used to evaluate the in vivo efficacy levels of the ZV54 variants. As previously described [44], the mice were divided into two groups (*n* = 10 per group) and inoculated subcutaneously with 1 × 10^5^ PFUs of ZIKV on the ventral sides of their right hind footpads. Twenty-four hours post-infection, the mice in group 1 were intraperitoneally treated with 280 µg of pZV54^WT^ mAb and the mice in group 2 (control group) received an equivalent volume of PBS. Blood was collected in Trizol on day 3 post-infection to quantitate the level of viremia by RT-qPCR. The survival of the mice was monitored daily for 20 days post-ZIKV infection.

### 2.11. Real Time-Quantitative PCR (RT-qPCR)

RT-qPCR was performed per the previously descripted procedure [44]. Briefly, TRI-reagent (Molecular Research Center Inc., Cincinnati, OH, USA) was used to isolate the total RNA from the mouse blood and an iSCRIPT cDNA synthesis kit (Bio-Rad, Hercules, CA, USA) was employed to synthesize the first-strand complementary DNA (cDNA) from the total RNA. The RNA copy numbers of the ZIKV E protein and cellular *β*-actin were then determined by RT-qPCR using the previously described primers [45] in a CFX96 Real-Time system (Bio-Rad, Hercules, CA, USA). The viral copy numbers were expressed as the PFU equivalent per milliliter of blood. Serial dilutions of the ZIKV PRVABC59 stock virus of a known titer were used to infect the Vero cells to generate the “standard” RNA samples. The PFU equivalents were determined by comparing the experimental RT-qPCR cycle threshold values (CT) to that of a standard curve generated by using the “standard” RNA samples.

### 2.12. Statistical Analyses

Statistical analyses were performed with GraphPad Prism software version 9.3. One-way ANOVA and T-tests were used, respectively, to compare the ADCC activities between the different mAb variants and the blood viral copy numbers between the mouse groups treated with pZV54^WT^ mAb and PBS. A *p* value of <0.05 indicated a statistically significant difference.

## 3. Results

### 3.1. Production of ZV54 mAb in Nicotiana benthamiana Plants

Six-week-old *N. benthamiana* plants were used to produce ZV54 mAb via transient expression. Our ELISA results indicated that ZV54 quickly accumulated in the leaves of both the WT and glycoengineered ΔXF *N. benthamiana* plants [26], with peak expression at 7 DPI (Figure S1). The correct assembly of ZV54 mAb was also verified by the same experiment as our ELISA only detected the fully assembled form of ZV54 mAb [46]. The plant- and CHO-cell-derived ZV54 mAbs were purified to homogeneity for further characterization using a purification method that we developed previously [34].

### 3.2. N-Linked Glycan Analysis of ZV54 mAb Produced in Different Hosts

Since the composition of glycans affects IgG’s Fc effector functions [47], LC-ESI-MS was used to characterize the N-glycosylation of the ZV54 produced in the various hosts. The WT plant-produced ZV54 (ZV54^WT^) carried complex-type N-glycans with N-acetylglucosamine terminal residues, xylose, and 1,3-linked core fucose (GnGnXF_3_), and the ZV54 expressed in the ∆XF plants (ZV54^∆XF^) exhibited the mammalian type GnGn structures that lacked xylose and fucose (Table 1). Remarkably, both ZV54^WT^ and ZV54^∆XF^ showed a single N-glycoform with 100% homogeneity. In contrast, the CHO cell-derived ZV54 (ZV54^CHO^) exhibited a heterogeneous glycosylation pattern of four structures with two major peaks of core α1,6 fucosylated structures with or without terminal β1,4-galactose (GnGnF_6_ and AGnF_6_) (Table 1).

### 3.3. Specific Binding of ZV54 Glycovariants to the Target Antigens

The specific recognition of the ZV54 glycovariants to the ZIKV E DIII was characterized in a binding assay with yeast that displayed zDIII on their surface, with zDIII expression under the control of an inducible promoter [48]. Flow cytometric analysis showed that only the basal levels of the fluorescence signals were detected when the uninduced yeast samples were stained with the ZV54 variants (Figure S2A). In contrast, a new fluorescent peak beyond the background signals was detected by the ZV54 variants when the yeast cells were induced for zDIII expression 24 h before staining (Figure S2B). ZV54^WT^ and ZV54^∆XF^ showed similar binding activities as their percentages of positive yeast samples and their mean fluorescence intensities of binding were nearly identical (Figure S2B). The specificity of the binding was further confirmed as E16, an mAb that can recognize the equivalent region of EDIII of WNV [37], failed to recognize the zDIII displayed on the yeast (Figure S2B). Therefore, the plant-produced ZV54 glycovariants retained the specific recognition of their target antigens.

### 3.4. ZV54 Glycovariants Potently Neutralize ZIKV

**Figure 1:**
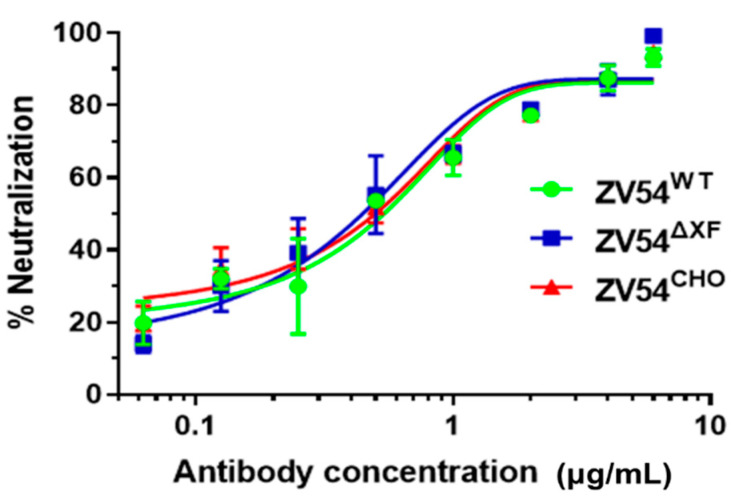
ZIKV neutralization by the ZV54 mAb glycovariants. The ZV54 mAb glycovariants were serially diluted and co-incubated with ZIKV (100 PFU) and Vero cells. Plaques were counted after 3 days of incubation, and the percent neutralization and IC50 were calculated with GraphPad. The experiments were repeated at least twice, with technical triplicates. The error bars represent the SDs of the means.

After confirming the specific binding of the ZV54 variants to zDIII, we examined the potential of the ZV54 glycovariants in neutralizing ZIKV by a PRNT assay. As shown in Figure 1, both ZV54^WT^ and ZV54^∆XF^ exhibited potent neutralizing activity against ZIKV, similar to that of ZV54^CHO^ (*p* > 0.99, for both ZV54^WT^ and ZV54^∆XF^ compared to ZV54^CHO^), with IC_50_ values of 1.97, 0.86, and 2.01 µg/mL for ZV54^WT^, ZV54^∆XF^, and ZV54^CHO^, respectively (Figure 1). These values represented the neutralization potency of ZV54 against a highly virulent American ZIKV strain that has not been reported in other studies [8].

### 3.5. Antibody-Dependent Enhancement (ADE) of DENV and ZIKV Infection by the ZV54 Glycovariants

**Figure 2:**
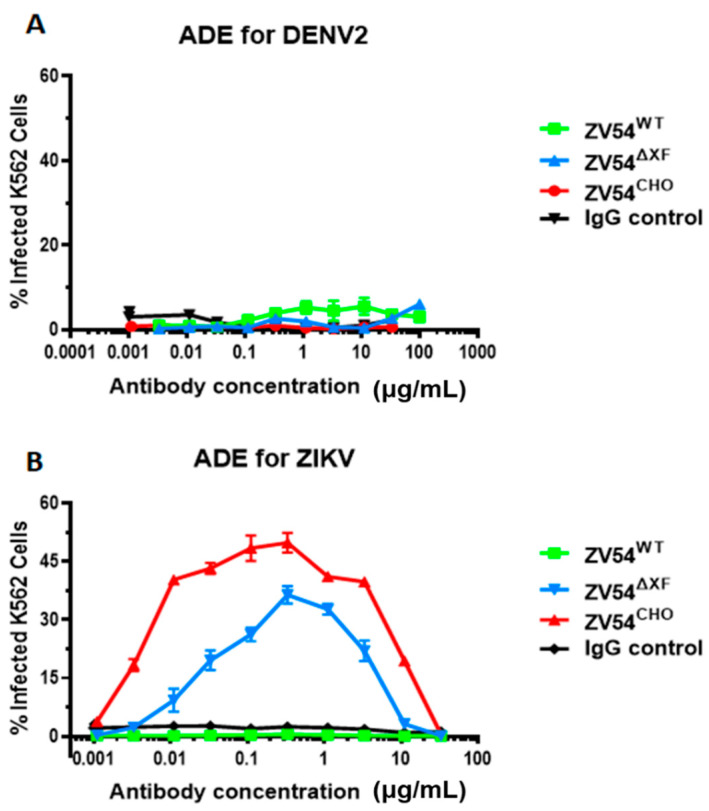
ADE activity of the ZV54 mAb glycovariants for DENV and ZIKV infection. DENV-2 (**A**) or ZIKV (**B**) samples were mixed with serial dilutions of the ZV54 mAb glycovariants or a generic IgG negative control and then incubated with FcγRIIa^+^ K562 cells. The cells were then washed, fixed, permeabilized, and stained with an anti-flavivirus E antibody. The DENV-2 or ZIKV infected cells were identified by flow cytometry. The experiments were performed at least twice, with technical triplicates for each sample of the presented results (means ± SDs).

Next, all three mAb glycovariants were investigated for their ability to induce the ADE of DENV and ZIKV infection. Since ZV54 was selected for its unique specificity to ZIKV [8], none of three glycovariants showed any activity in promoting ADE for DENV infection, similar to the IgG negative control (Figure 2A). For ZIKV infection, ZV54^CHO^ triggered a significant level of ADE (Figure 2B) in the FcγRIIa-expressing K562 cells. Interestingly, the two plant-derived ZV54 samples displayed drastically different ADE activities for ZIKV: ZV54^∆XF^ showed strong ADE activity but ZV54^WT^ completely lacked any ADE activity, similar to the IgG negative control (Figure 2B).

### 3.6. ADCC Activity of ZV54 Glycovariants

**Figure 3:**
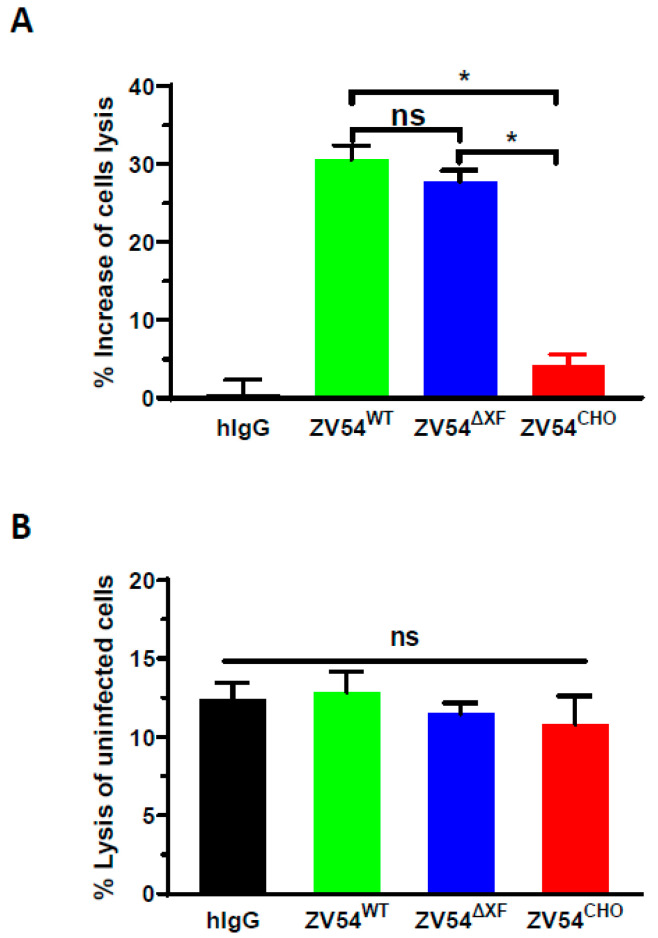
NK cell-mediated ADCC activity of the ZV54 mAb glycovariants. The ZIKV-infected Vero cells (**A**) or uninfected Vero cells (**B**) were incubated with NK cells (E:T ratio = 5:1) in the presence of the ZV54 glycovariants or an IgG isotype negative control (hIgG). The percent of Vero cell lysis (mAb–NK cell-mediated ADCC target-cell killing) and the percent increase in ZV54 ADCC compared to the negative control hIgG were calculated. * and ns indicate *p* values of <0.05 and >0.05, respectively.

Since Fc effector function such as ADCC is required for the full protective activity of anti-ZIKV antibodies in vivo [49], an NK-mediated ADCC assay was performed. All three ZV54 variants showed higher NK-mediated ADCC activity with the ZIKV-infected Vero cells than with the isotype control antibody (hIgG) (Figure 3A). Both plant-derived ZV54 glycovariants displayed enhanced ADCC activity with statistical significance compared to ZV54^CHO^ (Figure 3A, ZV54^∆XF^ vs. ZV54^CHO^: *p* = 0.044; ZV54^WT^ vs. ZV54^CHO^: *p* = 0.043). The specificity and ADCC dependency for the lysis of the ZIKV-infected cells were confirmed by parallel experiments with non-infected Vero cells, and no significant differences (*p* > 0.05) in cell lysis were observed, regardless of whether the negative control hIgG or any of the three glycovariants was used (Figure 3B).

### 3.7. Differential Binding of the ZV54 Glycovariants to Human FcγR Receptors

As human FcγRIIIA plays an important role in NK-mediated ADCC activity [50] and is also implicated in mediating the ADE of DENV infection, along with the major ADE-inducing FcγRIIA [51,52,53], we investigated the binding of ZV54 glycovariants to these FcγRs by SPR. ZV54^WT^ exhibited reduced binding to FcγRIIA (2.58 × 10^−6^ M), with an approximately four times lower affinity compared to ZV54^CHO^ (6.62 × 10^−7^ M) (Table S1). ZV54^∆XF^ also displayed reduced affinity to FcγRIIA but to a lesser degree (1.89 × 10^−6^ M) (Table S1). Regarding FcγRIIIA binding, the affinities were in the following order: ZV54^∆XF^ (4.38 × 10^−8^ M) > ZV54^CHO^ (7.40 × 10^−8^ M) > ZV54^WT^ (1.63 × 10^−7^ M) (Table S1).

### 3.8. Therapeutic Activity of the ZV54 Glycovariants against ZIKV Infection in Mice

**Figure 4:**
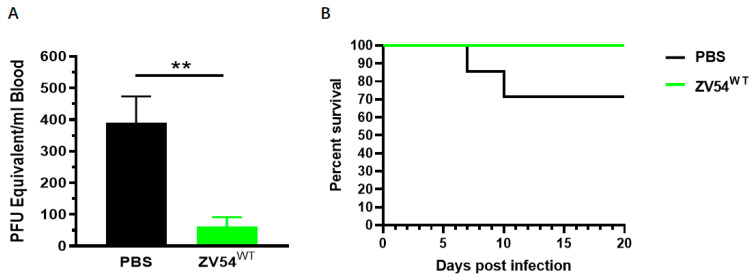
In vivo activity of the plant-made ZV54 glycovariants in mice. Five-weeks-old *Ifnar1*^−/−^ mice were inoculated with ZIKV and administered ZV54WT or PBS (negative control) intraperitoneally 24 h after ZIKV infection. Blood was collected on day 3 post-infection, and viremia was quantitated by RT-qPCR (**A**). Mice were further monitored daily for survival to 20 days post-infection (**B**). The results are representative of two independent experiments. ** indicates a *p* value of 0.0065.

The in vivo efficacy of ZV54^WT^ was tested in a mouse model as this glycovariant appears to preserve ADCC activity while forgoing ADE for both DENV and ZIKV infection. Five-week-old *Ifnar1*^−/−^ mice were divided into two groups, and both groups were infected with ZIKV via footpad inoculations. Twenty-four (24) hours post-infection, the mice in group 1 were treated with ZV54^WT^ (280 μg) while the mice in group 2 were administered with an equivalent volume of PBS. The RT-qPCR analysis indicated that the mice treated with ZV54^WT^ developed significantly lower viremia peaks at 3 days post-infection compared to the PBS-injected mice (*p* = 0.0065) (Figure 4A). Furthermore, 100% of the mice treated with ZV54^WT^ survived the ZIKV challenge compared to 70% for the mice that received PBS (Figure 4B). This result demonstrated that while forgoing ADE activity, the plant-derived ZV54^WT^ glycovariant retained the in vivo therapeutic efficacy against ZIKV infection.

## 4. Discussion

The effectiveness of mAbs against the E protein of ZIKV has been demonstrated to neutralize ZIKV, protecting against lethal ZIKV challenge in vivo, decreasing the transmission of ZIKV from mother to fetus, and preventing microcephaly in ZIKV mouse models [54,55,56]. Therefore, they are considered the leading candidates for treating ZIKV-infected patients. However, the potential risk of exacerbating diseases through ADE in subsequent DENV infections has hindered their clinical application.

In this study, we tested our hypothesis that using non-cross-reactive mAbs combined with antibody Fc glycoengineering is an effective approach to doubly secure the elimination of ADE while preserving the beneficial Fc effector functions that are necessary for the full potency of anti-ZIKV antibodies [57,58]. Our dual-approach strategy is based on the hypothesis that Fab-viral surface antigens and Fc-host-cell–FcγR interactions are both required for ADE to occur [59,60,61]. Therefore, using non-cross reactive mAbs and interrupting mAb binding to FcγRs may reduce or even eliminate the risk of ADE. Currently, the state-of-the-art for this purpose is performing Fc backbone mutation to fully abolish Fc–FcγR interactions [55,62] so that the risk of ADE is abrogated, along with other effector functions. Here, we used glycoengineering as an approach to fine-tune the Fc–FcγR interaction, hoping to identify antibody glycoforms that forgo ADE while retaining beneficial effector function.

It is possible to simultaneously evaluate the impact of both Fab specificity and Fc–FcγR modulation by characterizing the activity of glycovariants for a non-cross-reactive mAb that share an identical peptide backbone but with distinct Fc glycosylation profiles, as it has been well-described that Fc glycosylation strongly impacts the interactions of antibodies with host-cell FcγRs [63,64]. Accordingly, we produced such glycovariants for ZV54, a non-cross-reactive mAb, using glycoengineered plants that can produce mAbs with defined and homogeneous N-linked glycans [65]. Indeed, the plant-produced ZV54^WT^ and ZV54^∆XF^ exhibited largely homogeneous glycosylation profiles with single glycan species (GnGnXF3 and GnGn, respectively), while the CHO-derived parent ZV54^CHO^ carried at least four thereof, complicating the determination of the effects of glycan variation. Our results showed that variations in Fc glycosylation did not change either the antigen-binding specificity or the neutralization activity of ZV54, which was consistent with previous observations [66,67]. However, variations in N-glycosylation did result in significant differences in the abilities of the ZV54 glycovariants in triggering ADE for ZIKV. As observed for other anti-flavivirus antibodies of mammalian origin [68,69], ZV54^CHO^ displayed robust ADE activity. Similarly, ZV54^∆XF^ also exhibited ADE activity, similar to ZV54^CHO^. Strikingly, no ADE activity in ZIKV infection was observed for ZV54^WT^. The differences in ADE activity among the three ZV54 variants was most likely due to the variations in the Fc N-glycans because they had identical protein backbones.

One of the interesting remaining questions was whether any of the ZV54 glycovariants that forgo ADE still retain Fc effector function. Notably, NK-cell-mediated ADCC activities against ZIKV infection were observed for all three glycovariants, with the plant-produced variants having higher activities and statistical significance. Most importantly, ZV54^WT^ retained its ADCC activity while forgoing ADE. Significantly, we showed the in vivo activity of ZV54^WT^ in a relevant *Ifnar1*^−/−^ mouse model, demonstrating the potential of this ADE-free mAb variant as a post-exposure ZIKV therapeutic.

The mechanism for a particular Fc N-glycosylation such as GnGnXF_3_ to reduce ADE while preserving ADCC activity is unclear and requires more studies to elucidate. One interpretation of our results would suggest that ADE and ADCC may be mediated by different FcγRs. For example, the exhibition of ADCC activity but the lack of ADE suggested that ZV54^WT^ retained some affinity to ADCC-mediating FcγRIIIA but has reduced binding to ADE-causing FcγRs, such as FcγRIIA. Indeed, our SPR results corroborated such a hypothesis, as ZV54^WT^ displayed reduced—yet still strong—binding to FcγRIIIA and reduced binding to FcγRIIA, which has been implicated as a major FcγR in mediating DENV ADE [53,60,70]. However, in contrast to the above interpretation, some studies have also suggested a possible role of FcγRIIIA for DENV ADE induction [51]. Since K562 cells do not display FcγRIIIA, the effect of glycan variation on such a proposed ADE mechanism cannot be measured by this extensively used but imperfect in vitro ADE system. Based on our SPR results, however, we can speculate that ZV54^WT^ may also reduce FcγRIIIA-mediated ADE as the affinity of ZV54^WT^ to FcγRIIIA was reduced to a similar degree as that of FcγRIIA, which effectively abrogated FcγRIIA-mediated ADE. However, it is important to note that the induction of ADE requires more than only the Fc-mediated binding of a virus–antibody complex to FcγRs as the downstream signaling post-FcγR-binding has been shown to be critical for ADE to occur [71,72]. For example, FcγRIIA and FcγRIIB share similar Fc-binding ectodomains but have distinct intracellular signaling domains. As a result, the binding of the DENV-immune complex to FcγRIIA induces ADE but the same binding to FcγRIIB inhibits this activity [52,72,73]. Therefore, it is possible that N-glycan variation may impact both the binding of immune complexes to FcγR-expressing cells and the downstream signaling that is required for viral entry and replication. Although we cannot extract a final mechanistic conclusion, it appears that the specific FcγR binding signature of ZV54^WT^ has a direct impact on the preservation of ADCC and the abrogation of ADE. These remaining questions that cannot be answered by the in vitro system warrant future animal experiments with human FcγR-expressing mouse models and with more detailed analyses such as in vivo ADE and effector function measurement to reveal the full effects of ZV54 Fc glycoengineering.

The imperfection of the current K562-based in vitro ADE system also emphasized the importance of our dual-approach strategy in securing the safety of anti-flavivirus therapeutics. Within these two approaches, the modulation of FcγR binding by Fc glycoengineering provides a strategy with broad impacts as it has the potential to simultaneously reduce/eliminate ADE by multiple related viruses and it can be applied to both virus/serotype-specific and broadly neutralizing mAbs [74]. This strategy is highly valuable in developing pan-flavivirus mAb therapeutics and treating diseases when a broadly neutralizing mAb alone or an mAb cocktail is required. This strategy also provides a significant advantage over the current strategy of Fc-silenced mutations where ADE abrogation is accompanied by the loss of important Fc effector functions. In contrast, selecting virus/serotype-specific mAbs presents a narrower yet necessary strategy in circumstances where Fc effector function is required for protection, and current technology in Fc glycoengineering cannot ensure the full abrogation of ADE. For example, the lack of ADE in the DENV infections for all three ZV54 glycovariants due to the high specificity of this mAb [8] validated the importance of selecting virus/serotype-specific mAbs as a strategy to avoid ADE and the associated severe DENV symptoms that have been demonstrated clinically. Even though there is no mechanistic connection between Fc glycosylation and Fab specificity, our dual-approach strategy presented an effective way to avoid ADE, especially under the current circumstances where there are no optimal in vitro or in vivo models for testing the ADE mediated by all relevant FcγRs on various immune cells. Further optimization of Fc glycoengineering is warranted to identify glycoforms that may reduce/eliminate the ADE mediated by all relevant FcγRs.

ZV54^WT^, which abrogated ADE but preserved both the in vitro and in vivo efficacies, carries complex N-glycans containing β1,2-xylose and α1,3-fucose, which are more abundant in glycoproteins of plant origin [65]. This may raise concerns for the potential unintended immune responses in humans that may cause adverse health risks. However, all results from the clinical trials of plant-made biologics to date have shown that the presence of such sugar residues does not induce any unwanted side effects [75,76]. In recent decades, mAbs against a diverse array of targets have been produced in plants [77]. Some of these mAbs have been produced under current Good Manufacturing Practices (cGMP) as proof-of-principle that plants can be a viable alternative to mammalian-made biologics [78,79,80]. Studies have demonstrated that plants offer a more cost-effective production system compared to CHO-based platforms while also greatly reducing the risk of contamination by mammalian viruses or other pathogens [81,82]. Therefore, the development of plant-derived ZV54 glycovariants may also contribute to the improvement in affordability for mAb-based therapeutics in the developing world, where the majority of ZIKV cases exists.

Overall, we have reported a dual-approach strategy for increasing the safety and efficacy of anti-ZIKV mAb therapeutics. The use of non-cross-reactive mAbs against ZIKV eliminates the risk of ADE in enhancing the symptoms of infection by related flaviviruses, such as DENV. The identification of an mAb glycoform that forgoes ADE while maintaining effector function further increases the efficacy and safety of anti-ZIKV mAbs as this strategy also prevents even the theoretical risk of ADE by a newer variant of ZIKV. The impacts of our findings may reach beyond the flavivirus disease model as this approach can be applied to the development of safer mAb-based therapeutics for other ADE-prone viruses, including SARS-CoV-2.

## Figures and Tables

**Table 1:** N-glycosylation profile of the ZV54 mAb variants. Purified ZV54^CHO^, ZV54^WT^, and ZV54^ΔXF^ were separated by SDS-PAGE and their heavy chains were excised, trypsin-digested, and analyzed by LC-ESI-MS. The percentage of each N-glycoform was assigned based on the approximate molar ratio of the height of the corresponding glycopeptide peak. The glycoforms were annotated based on the nomenclature of the Consortium for Functional Glycomics.

Major N-Glycan Species (in %)	Schematic Presentation	ZV54^CHO^ (%)	ZV54^WT^ (%)	ZV54^ΔXF^ (%)
**GnGn**	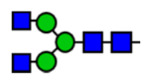			100
**GnGnXF_3_**	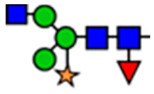		100	
**GnGnF_6_**	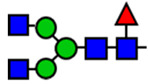	68		
**MGnF_6_**	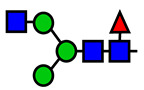	6		
**AGnF_6_**	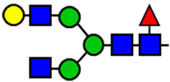	22		
**AAF_6_**	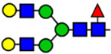	4		

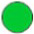
: Mannose, 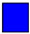: N-acetylglucosamine, 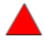: Fucose, 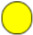: Galactose, 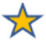: Xylose.

## Data Availability

The data presented in this study are contained within this article.

## Supplementary Materials

The following supporting information can be downloaded at: https://www.mdpi.com/article/10.3390/v15051156/s1, Figure S1: Expression of ZV54 mAb in N. benthamiana plants; Figure S2: Specific binding of plant-derived ZV54 glycovariants to zDIII displayed on yeast surface; Table S1: Binding of ZV54 mAb variants to human FcγR receptors.
