# Supplementary material for: A Dual-Approach Strategy to Optimize the Safety and Efficacy of Anti-Zika Virus Monoclonal Antibody Therapeutics
Source: Viruses. 2023 May 11;15(5):1156. doi: 10.3390/v15051156 (PMC10221487; doi:10.3390/v15051156)
Supplement: Supplementary file 1 [file viruses-15-01156-s001.zip › viruses-2311349-supplementary.pdf]

Fig S1

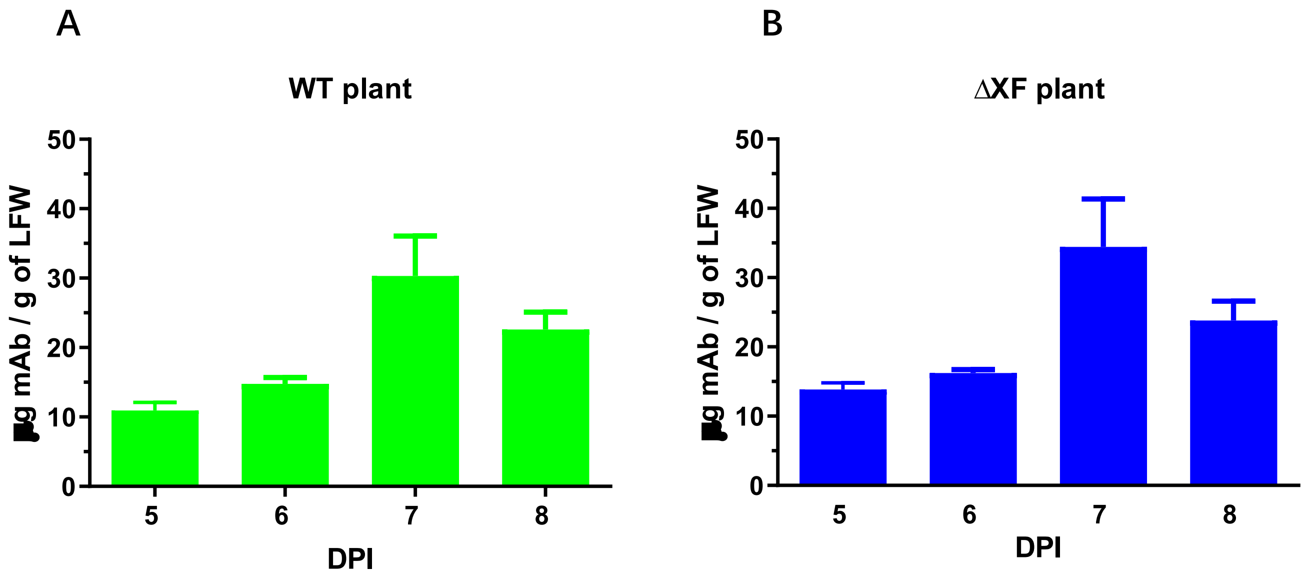

Figure S1. Expression of ZV54 mAb in *N. benthamiana* plants.
Total soluble protein was extracted from WT (**A**) or ΔXFT (**B**) *N. benthamiana* leaves that were agroinfiltrated with vectors carrying the expression cassette of ZV54 mAb light chain and heavy chain. Levels of mAb accumulation 5-8 days post infiltration (DPI) were measured by an ELISA that only detects fully assembled IgG. Results (Mean ± SD) are from three independent infiltrations with technical triplicates for each sample.

Fig S2

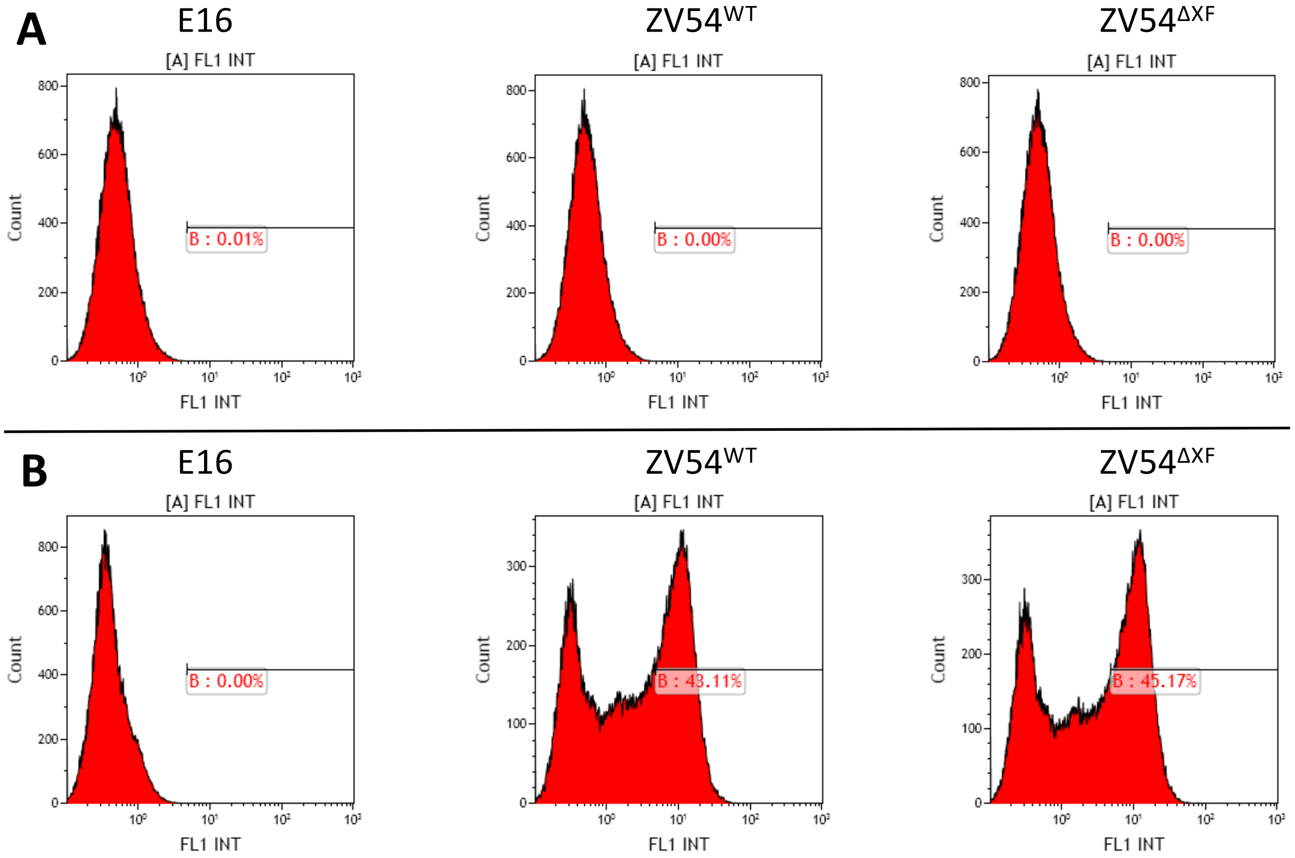

Figure S2. Specific binding of plant-derived ZV54 glycovariants to zDIII displayed on yeast surface.

Yeast cells were either uninduced (**A**) or induced for 24 hr (**B**) for surface zDIII expression and then stained with $ZV54^{WT}$, $ZV54^{\Delta XF}$ or a negative control mAb (E16, binds to the equivalent region on DIII of WNV). Stained yeast cells were processed by flow cytometry. Representative data from several independent experiments are shown.

Table S1

	FcγRIIA (K_D)	FcγRIIIA (K_D)
**ZV54^{WT}**	**2.58E-6**	**1.63E-7**
**ZV54$^{\Delta XF}$**	**1.89E-6**	**4.38E-8**
**ZV54^{CHO}**	**6.62E-7**	**7.40E-8**

Table S1. Binding of ZV54 mAb variants to human FcγR receptors.

SPR was performed to determine the equilibrium dissociation constants (K_D) of ZV54^{WT}, ZV54$^{\Delta XF,}$ and ZV54^{CHO} to human FcγRIIA and FcγRIIIA. ZV54 variants were captured by CM5 chips that were coated with Protein A. Serial dilutions of recombinant FcγRs were then injected over the captured ZV54 variants. Binding responses were analyzed with BIAcore Evaluation software to calculate the value of K_D (M).
